# Herbal Medicines Attenuate PD-L1 Expression to Induce Anti-Proliferation in Obesity-Related Cancers

**DOI:** 10.3390/nu11122979

**Published:** 2019-12-05

**Authors:** Yu-Chen S.H. Yang, Zi-Lin Li, Ya-Jung Shih, James A. Bennett, Jaqueline Whang-Peng, Hung-Yun Lin, Paul J. Davis, Kuan Wang

**Affiliations:** 1 Joint Biobank, Office of Human Research, Taipei Medical University, Taipei 11031, Taiwan; can_0131@tmu.edu.tw; 2 Graduate Institute of Nanomedicine and Medical Engineering, College of Medical Engineering, Taipei Medical University, Taipei 11031, Taiwan; lizilin0919@gmail.com (Z.-L.L.); shihyj@tmu.edu.tw (Y.-J.S.); jqwpeng@nhri.org.tw (J.W.-P.); wangk007@gmail.com (K.W.); 3 Taipei Cancer Center, Taipei Medical University, Taipei 11031, Taiwan; 4 Center for Immunology and Microbial Diseases, Albany Medical College, Albany, NY 12208, USA; bennetj@mail.amc.edu; 5 Cancer Center, Wang-Fan Hospital, Taipei Medical University, Taipei 11031, Taiwan; 6 Graduate Institute of Cancer Biology and Drug Discovery, College of Medical Science and Technology, Taipei Medical University, Taipei 11031, Taiwan; 7 TMU Research Center of Cancer Translational Medicine, Taipei Medical University, Taipei 11031, Taiwan; 8 Traditional Herbal Medicine Research Center of Taipei Medical University Hospital, Taipei Medical University, Taipei 11031, Taiwan; 9 Pharmaceutical Research Institute, Albany College of Pharmacy and Health Sciences, Rensselaer, NY 12208, USA; pdavis.ordwayst@gmail.com; 10 Department of Medicine, Albany Medical College, Albany, NY 12208, USA

**Keywords:** thyroid hormone, steroid hormone, resveratrol, curcumin, anoectochilus formosanus hayata, PD-L1

## Abstract

Pro-inflammatory hormones and cytokines (leptin, tumor necrosis factor (TNF)-α, and interleukin (IL)-6) rise in obesity. Elevated levels of hormones and cytokines are linked with several comorbidities such as diabetes, heart disease, and cancer. The checkpoint programmed cell death protein 1 (PD-1)/programmed death-ligand 1 (PD-L1) plays an important role in obesity and cancer proliferation. L-thyroxine (T_4_) and steroid hormones up-regulate PD-L1 accumulation and promote inflammation in cancer cells and diabetics. On the other hand, resveratrol and other herbal medicines suppress PD-L1 accumulation and reduce diabetic effects. In addition, they induce anti-cancer proliferation in various types of cancer cells via different mechanisms. In the current review, we discuss new findings and visions into the antagonizing effects of hormones on herbal medicine-induced anti-cancer properties.

## 1. Background

The programmed death (PD)-1/PD-ligand 1 (PD-L1) checkpoint is a crucial modulator of the interactions between triggered T-cells and tumor cells. The checkpoint defends tumor cells against immune destruction. PD-L1 (or B7-H1) is a protein produced by cancer cells that interacts with PD-1 and suppresses activated T-cell from engaging with cancer cells. PD-L1 ligand activity may also induce apoptosis of T-cells. PD-L1 overexpression is observed in melanoma, pancreatic, lung, and other types of cancer cells. This situation may be associated with reduced patient survival. Recently, success with immunotherapy has shifted the cancer treatment paradigm, however, because only a small group of patients respond to immunotherapy, it is crucial to identify the factors that influence outcomes. In addition, obesity is approaching pandemic proportions worldwide. Obesity is also a main risk factor for some specific malignancies. However, the general impacts of obesity on immune responses and on cancer immunotherapy are incompetently explored [1]. Obesity, defined by a gained body-mass index (BMI of ≥30 kg/m^2^), reflects visceral fat accumulation. There is also an increase in secretion of pro-inflammatory hormones such as the thyroid hormone and cytokines (leptin, tumor necrosis factor (TNF)-α, and interleukin (IL)-6) in obesity. On the other hand, obesity may reduce the release of anti-inflammation adipokines such as adiponectin and IL-10 [2]. Obesity is linked with numerous comorbidities such as diabetes, heart disease, and cancers. Additionally, inflammation and oxidative stress also play a role in various disease processes, including diabetes, cardiovascular diseases, and cancers. Moreover, obesity characterizes a substantial societal burden, accounting for  >20% of the total annual US healthcare expenditure. In this current review, we discuss the impacts of thyroid hormone, L-thyroxine, and steroid hormones on inducing PD-L1 in obesity complications, such as cancer. The antagonist effects of herbal medicines on PD-L1 expression are also addressed. The interactions between herbal medicines and hormones are emphasized.

## 2. Obesity, PD-L1, and Cancers

Obesity represents a low-grade chronic inflammatory state, which may damage cell-mediated immunity. It may also increase the risk of infections. Obesity is also a significant risk factor for colorectal cancer and other malignancies. In fact, obesity disturbs T-cell generation and functions. Consequentially, it impairs the ability to activate peripheral T-cell-mediated protective immune responses [3,4]. Hyperglycemia stimulates the production of reactive oxygen species (ROS) and promotes oxidative stress. ROS and oxidative stress increase risk for cancer progression and metastasis [5,6,7]. Additionally, adipocytes produce inflammatory mediators to tremendously stimulate inflammation [5]. Overweight people may have a type of tumorigenic immune dysfunction, which can be effectively reversed by immune checkpoint inhibitors. BMI is useful to predict whether a patient is overweight in clinical practice. It is a stratification factor in prospective clinical trials with immune checkpoint inhibitors [8].

Even though obesity as a ‘meta-inflammatory’ state presents dysregulated immune responses and ‘inflammaging’ [1], its impact on immune responses during cancer progression and immunotherapy is not fully clarified. In pre-clinical studies, young and lean mice are used to study cancer progression and development to represent elderly patients with cancers [1]. However, most studies fail to present the clinical situations of cancer patients. Although no clear mechanisms have been elucidated, clinical analyses indicate that obesity is linked with better outcomes and patient survival in cancer treated with targeted therapy and checkpoint blockade immunotherapy [9].

Negative checkpoint regulators reduce immune responses to inhibit immune activation, decrease collateral damage, and sustain peripheral self-tolerance [10]. Two of the most intensively studied negative checkpoint regulators are cytotoxic T lymphocyte (CTL)-associated antigen 4 (CTLA-4, also called cluster of differentiation 152 (CD152)) and PD-1 (also called CD279) [11]. They modulate immune responses differently at completely different levels. Primarily, CTLA-4 regulates the amplitude of the early stages of T-cell activation. Alternatively, PD-1 mainly regulates effector T-cell activity within tissues and tumors, where the immune response is ongoing [12]. PD-L1 is involved in evading immune surveillance [13]

The B- and T-lymphocyte attenuator (BTLA) is a checkpoint co-inhibitory receptor categorized into the CD28 superfamily (immunoglobulin (Ig) superfamily) [14]. It is present in a large range of immune cells, including T-cells, B cells, and natural killer (NK) cells. BTLA is structurally and functionally related to CTLA-4 and PD-1 [15,16]. An augmented BTLA level is associated with the progress and poor prediction of gastric cancer [17]. In addition, PD-L1 overexpression was shown to interfere with cell cycle, cell growth, apoptosis, and carcinogenesis [18]. Thus, checkpoint gene overexpression may modulate cancer growth and progression.

Recently, results from our group indicate that treatment of cancer cells with the extract of *Anoectochilus formosanus* Hayata, a traditional anti-inflammatory herbal medicine, inhibited constitutively expressed *PD-L1* and protein accumulation. Furthermore, it also inhibited cancer proliferation [7]. These observations suggest that PD-L1 may be involved in an inflammatory effect on cancer proliferation [7]. An obvious effect of obesity on tumor progression in a mouse model and on clinical outcomes in cancer patients treated with a PD-1/PD-L1 checkpoint blockade was based on body mass [8]. Those studies pointed out consistent effects of obesity on cancer immune responses in an immunotherapy context. Therefore, PD-L1 may have supplementary functions in tumor cells that are independent of the checkpoint to induce cancer survival.

Interferon (IFN)-γ and epidermal growth factor (EGF) are two endogenous inducers of PD-L1 expression. Evidence also indicates that pro-inflammatory cytokines such as TNF-α [19] and IL-1 [20] can induce PD-L1 expression. TNF-α may enhance IFN-γ-induced PD-L1-mediated adaptive immune resistance in hepatocellular carcinoma cells [21].

Receptor-mediated signaling pathways play vital roles in PD-L1 induction. Nuclear factor (NF)-κB [22], phosphoinositide 3-kinase (PI3K) [23,24], extracellular signal-regulated kinase-1 and -2 (ERK1/2) [23,24], Janus kinase/signal transducer and activator of transcription (JAK/STAT) [25,26], and mammalian targets of rapamycin (mTOR) are shown to be involved in PD-L1 expression in tumor cells. Estrogen upgrades PD-L1 protein accumulation via the activated PI3K/Akt pathway in Ishikawa cells and human breast cancer MCF-7 cells. Inhibitors of PI3K and Akt attenuate estrogen’s effects [27]. The activated signal transducing pathways of ERK1/2, PI3K, and STAT3 are critical for the expression of thyroxine-induced PD-L1 in different types of cancer cells [28,29,30].

## 3. Thyroid Hormone and PD-L1

Inhibitors of immune checkpoints block the functions of checkpoint molecules. Several types of immune checkpoint inhibitors for cancer treatment have been approved recently—anti-PD-1 monoclonal antibodies (such as pembrolizumab and nivolumab); anti-PD-L1 monoclonal antibodies (such as atezolizumab); and CTLA-4 monoclonal antibodies (such as ipilimumab, avelumab, and durmalumab) [31]. The consequence is usually about 50% irreversible in immune-related endocrine toxicities. Those toxicities include hypophysitis, adrenal insufficiency, type 1 diabetes mellitus, and thyroid dysfunctions [31]. Particularly, hypophysitis is the most common anti-CTLA-4-antibody-related immune-related adverse event (irAE). On the other hand, thyroid abnormalities like thyrotoxicosis, hypothyroidism, painless thyroiditis, and even thyroid storms are more commonly related to applying anti-PD-1 antibodies [31].

Thyroxine induces the expression of *BTLA* and *PD-L1*, accompanied with the up-regulated expression of the proliferative gene Homo sapiens cyclin D1 (*CCND1*), and the coincidental down-regulated expression of the pro-apoptotic gene Homo sapiens BCL2-associated agonist of cell death (*BAD*) in cancer cells [32]. Structurally, BTLA is related to PD-1 and CTLA-4. There are an immune-receptor tyrosine-based inhibitory motif (ITIM) and an immune-receptor tyrosine-based switch motif (ITSM) in BTLA. Its ligand, HVEM (also called TNFRSF14), belongs to the TNF receptor (TNFR) superfamily. Generally, HVEM is present on hematopoietic cells and on a variety of parenchymal cells such as breast, melanoma, esophageal, colorectal, and ovarian cancer cells [32]. Interestingly, thyroxine was shown to stimulate cancer cell proliferation in those cancers and may play a role in HVEM-related immune surveillance. The binding of BTLA to the cysteine-rich domain 1 (CRD1) of HVEM makes this pathway necessary in the cross-talk between Ig and the TNF superfamily. Additionally, the BTLA/HVEM signal pathway seems to be a new possible approach of immune escape, which is considered a critical factor in inflammatory physiological processes. Thyroxine may play an important role in BTLA/HVEM-related inflammation and tumorigenesis.

The thyroid hormones, 3,5,3′-triiodo-L-thyronine (T_3_) and thyroxine, were shown to promote the growth of cancer cells [30,33,34,35,36]. Recent studies by our group also indicated that thyroxine stimulates the growth of human ovarian and lung cancer cells via the cross-talk between the cell surface αvβ3 integrin receptor and estrogen receptor α (ERα) [37,38]. Thyroxine induces ERK1/2 activation to induce *PD-L1* gene expression and consequent PD-L1 protein abundance in different cancer types [28,33]. Furthermore, the thyroid hormone was demonstrated to be involved in regulating oxidative stress [39]. Hyperthyroidism [40,41] increases reactive oxygen species (ROS), the most important pro-oxidants. Thyroxine can induce the expression of pro-inflammatory genes [42] to moderate inflammatory activities. The increased inflammation may correlate to cancer progression.

## 4. Steroid Hormone and PD-L1

In addition to the thyroid hormone, estrogen is able to up-regulate the accumulation of PD-L1 protein in ERα-positive endometrial and breast cancer cells [27]. Overexpression of PD-L1 suppresses T-cell immune functions in tumor microenvironments [27]. 1,25-Dihydroxyvitamin D (1,25D) is capable of directly inducing PD-L1 and PD-L2 expressions through the vitamin D receptor [43], suggesting that activated vitamin D signaling in humans can suppress antitumor immunity. Remarkably, 17β-estradiol does not up-regulate PD-L1 expression but instead stabilizes PD-L1 messages. In contrast, vitamin D and thyroxine increase PD-L1 expression. Furthermore, the effect of 17β-estradiol is only observed in ERα-positive Ishikawa and MCF-7 cells but not in ERα-negative MDA-MB-231 cells. Alternatively, thyroxine possibly induces PD-L1 expression through the integrin αvβ3 signal transduction pathway.

## 5. Herbal Medicines, Obesity, and PD-L1

### 5.1. Resveratrol

Resveratrol is a polyphenol that exists in different plants [44]. This antioxidant stilbene has been shown to have anti-inflammatory effects [45]. Thus it has been discovered to have cardiovascular protective effects [46], anti-cancer proliferative effects [47,48], and anti-diabetic effects [49]. Resveratrol can attenuate the expression of pro-inflammatory genes [50]. Regulatory T-cells (Tregs) are crucial negative regulators of inflammation [51]. Resveratrol reverts the damaging effects of T-cell function in diet-induced obesity [51] Additionally, resveratrol supplemented in a high-fat diet (HFD) relieved oxidative stress, inhibited inflammatory gene expressions, and increased regulatory Treg counts by activating the aryl hydrocarbon receptor in a mouse model of HFD-induced obesity [52]. Furthermore, resveratrol can activate the antioxidant enzyme expression mediated by nuclear factor erythroid 2-related factor 2 (Nrf2) [52]. Resveratrol inhibits inflammation by protecting against oxidative damage and subset T-lymphocyte-dependent chronic inflammatory responses in HFD-induced obesity animal models [52]. Activating the PI3K and Sirtuin 1 (Sirt1) signaling pathways by resveratrol can maintain glucose homeostasis [51]. Generally, in the clinic, resveratrol can be used to treat activated T-cell-induced inflammation and other T-cell-related diseases.

Resveratrol can also induce anti-proliferation in various cancer cells [33,34,47] and inhibit cancer growth in vivo [53]. Resveratrol activates ERK1/2 via binding to the receptor on αvβ3 integrin. ERK1/2 activation is crucial for resveratrol-induced *COX-2* expression and protein nuclear accumulation to inhibit cancer proliferation. The nuclear accumulated phosphorylated ERK1/2 and COX-2 complex with p53 to trigger phosphorylation of p53 at Ser-15, to induce p53-dependent gene expression and to induce anti-proliferation sequentially [29]. On the other hand, thyroxine also binds the cell surface integrin, αvβ3, to activate signal transduction and induce its proliferation.

Resveratrol was shown to attenuate T-cell activation and decrease cytokine creation. Irregular T-cell activation occurs in several autoimmune diseases such as insulin-dependent diabetes, rheumatoid arthritis, systemic lupus erythematosus, and multiple sclerosis [54]. It is likely that resveratrol is able to avert autoimmune disease progression. Studies indicated that resveratrol is able to block T-cell activation and antibody production in vivo [55]. Sirt1 activation mediates resveratrol-induced inhibition of T-cell activation. Resveratrol increases Sirt1 acetylase activity on c-Jun [55]. However, resveratrol does not increase the acetylation on NF-κB or the nuclear factor of activated T-cells (NFAT) in T-cells [55].

Nevertheless, resveratrol is unable to inhibit the acetylation of c-Jun in Sirt1-/- T-cells, dynamically indicating that the change of acetylation on c-Jun is totally dependent on Sirt1. c-Jun is translocated into the nuclei after T-cell activation. Nevertheless, resveratrol inhibits the action of c-Jun when T-cells are treated with resveratrol. Therefore, resveratrol increases Sirt1 expression and Sirt1 deacetylase activity on c-Jun to prevent c-Jun nuclear translocation sequentially [56]. This consequently blocks T-cell activation. Additionally, the activation of protein kinase Cθ may play a vital role in resveratrol-suppressed T-lymphocyte activation. Studies also showed that in a rat liver transplantation model, resveratrol inhibits protein kinase Cθ activity and T-lymphocyte activation in the peripheral blood T lymphocytes [57]. Conclusively, resveratrol targets T-cell activation in a bidirectional manner. It down-regulates CD4+ T-cell activation in autoimmune disease [56], while resveratrol diminishes the suppressive function of Tregs to inhibit tumor growth [58].

### 5.2. Curcumin

Curcumin (the extract of *Curcuma longa L.*) and its derivatives represent one of the most frequently used traditional medicines. They have been proved to have significant anti-inflammation and anti-oxidation both in vitro and in vivo [59,60,61]. Accordingly, curcumin potentially counteracts cancer-promoting inflammation and anti-diabetic activity [62]. Curcumin has been known to enhance immune responses through multiple mechanisms [63]. It inhibits the expression of pro-inflammatory genes to induce anti-inflammation. COP9 signalosome is a multifunctional regulator for Drosophila development [64]. NF-κB p65-induced COP9 signalosome 5 (CSN5) is required for TNF-α-mediated PD-L1 stabilization in cancer cells [64]. Curcumin was shown to inhibit CSN5 to diminish PD-L1 expression in cancer cells [64]. Treatment with curcumin inhibits the expression of PD-L1 and p-STAT3Y705 both in vitro and in vivo. Additionally, curcumin treatment can change the immunosuppressive tumor microenvironment. Studies have indicated that curcumin promoted an antitumor immune response effectively in tongue squamous cell carcinoma [65].

Bladder carcinomas induce the expression of PD-L1 to abolish the response of CD8 + T-cells. When it was used in combination with an α-PD-L1 antibody, bisdemethoxycurcumin, a naturally produced curcumin dimethoxy derivative, it was shown to reduce PD-L1 expression to provide a promising environment for T-cell responses against bladder cancer [66]. A combination of curcumin and apigenin suppresses cancer cell growth and induces pro-apoptotic effects in melanoma cells [67]. On the other hand, flavonoids, especially apigenin, inhibited IFN-γ-induced PD-L1 up-regulation via significantly inhibiting STAT1 phosphorylation [64]. In addition, combined treatment sensitizes cancer cells to anti-CTLA4 therapy [64].

## 6. *Anoectochilus Formosanus* Hayata

Golden thread (*A. formosanus* Hayata), a traditional herbal medicine, is used to treat various diseases including hyperglycemia. It is also an ROS scavenger. The *Anoectochilus formosanus* extract (AFE) inhibits the constitutive expression of *PD-L1* and accumulation of its protein [7]. AFE also induces the expression of pro-apoptotic genes but inhibits the expression of proliferative and metastatic genes. Therefore AFE induces anti-proliferation in cancer cells [7]. AFE reduced blood glucose concentrations as well as metformin. These results indicate the potential use of AFE in cancer immune chemoprevention/therapy through combining the mechanisms involved in producing a hypoglycemic effect, ROS scavenging, and *PD-L1* suppression.

## 7. The Balance between Herbal Medicines and Hormones in PD-L1 Expression

Thyroid hormones and steroid hormones induce ERK1/2 activation via the cell surface integrin, αvβ3, and specific steroid hormone receptors [33,35,37,47,68]. Thyroxine activates ERK1/2 to stimulate β-catenin-HMGA2-dependent proliferation [69]. Additionally, thyroxine promotes PD-L1 expression. Accumulated PD-L1 protein retains resveratrol-induced COX-2 in the cytosol in resveratrol and thyroxine co-treated cells. Resveratrol inhibits the thyroxine-induced proliferative effects in cancer cells by inhibiting the thyroxine-induced PD-L1 accumulation [36]. Nevertheless, the role of BTLA in thyroxine-induced pro-inflammatory actions and cancer proliferation is not yet well understood. Furthermore, resveratrol can attenuate estrogen- and androgen-induced proliferation of hormone-sensitive cancer cells.

Resveratrol can inhibit thyroid hormone-induced proliferation [28,47] and pro-inflammatory effects [30]. Both thyroxine and resveratrol bind to the αvβ3 cell surface integrin to activate ERK1/2. Resveratrol induces nuclear COX-2 accumulation and p53 phosphorylation via ERK1/2 activation to lead to COX-2-phosphorylated p53-dependent apoptosis. The treatment of resveratrol in cells induces PD-L1 retention in the cytosol and reduces thyroxine-induced PD-L1 nuclear accumulation under physiological conditions. Resveratrol stimulates COX-2 nuclear accumulation and COX-2-dependent anti-proliferation in dihydrotestosterone (DHT)-treated LNCaP prostate cancer cells, most likely via a similar mechanism as described in the model of resveratrol increasing inducible COX-2, which traps PD-L1 in the cytosol.

Curcumin induces a stimulatory effect on the thyroid gland’s secretory function in young rats, and has a rather weak anti-thyroid activity in old animals [70]. Treatment with curcumin significantly increases the levels of T_3_ and T_4_ in 3-month-old experimental rats [70]. Clinically, there is no interference in the effect between thyroid hormone replacement therapy and the curcumin formulation (Meriva^®^) (Milano, Italy) for osteoarthritis complementary treatment [71]. However, studies by Jena et al. indicate that hyperthyroidism affects curcumin-regulated superoxide dismutase (SOD) expression in different regions (cerebral cortex and cerebellum) of the rat brain [72]. Therefore, the effect of thyroxine on curcumin-induced activities needs more investigation in the future. Curcumin suppresses prostate cancer growth via the down-regulation of androgen receptors in androgen-sensitive prostate cancers [73], suggesting that androgens may not affect curcumin-induced biological functions.

AFE has a hypoglycemic effect in normal and diabetic mice [7]. Hyperglycemia usually accompanies the increased thyroid-stimulating hormone (TSH), triiodothyronine, and thyroxine levels [74]. AFE suppresses PD-L1 expression in the normal serum culture condition, which contains thyroid hormone and steroid hormones. AFE at concentrations of 0.2 mg/mL and 1 mg/mL blocked 28.6% and 100% PD-L1 expression, respectively [7]. These studies suggest that thyroid hormones and steroid hormones may not interfere with the activities of AFE.

## 8. Conclusions

**Figure 1:**
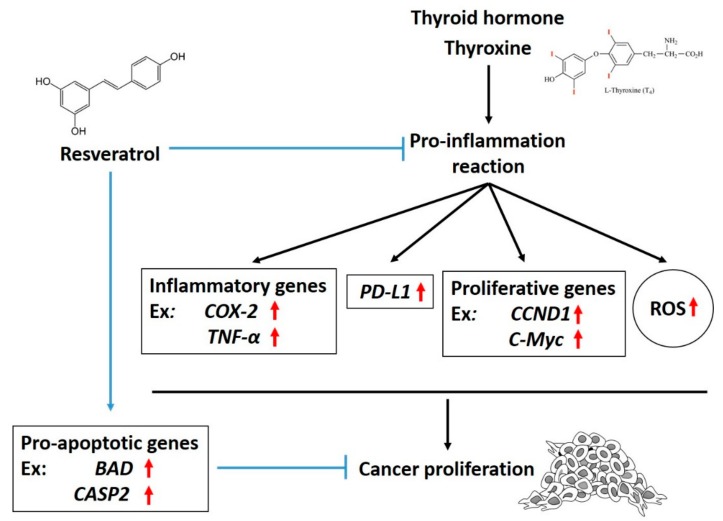
Mechanisms involved in the inhibitory effect of resveratrol on thyroxine-induced programmed cell death (PD)-1-dependent cellular activities in cancer cells. Thyroxine increases reactive oxygen species (ROS) accumulation and stimulates the expression of PD-ligand 1 (PD-L1) and inflammatory genes. Resveratrol inhibits PD-L1 expression and further blocks the expression of inflammatory, proliferative, and metastatic genes. All of these are important for the anti-proliferative effect against cancer cells. ↑: increased expression.

Obesity is a growing pandemic problem and is related to certain types of cancers. Recent research indicates that obesity affects immune responses, in general, and cancer immunotherapy. Steroid hormones and thyroxine were shown to play vital roles in PD-L1 expression. The increased accumulation of PD-L1 increases cancer resistance to anti-cancer therapies, especially in obese cancer patients. Several types of herbal medicines were shown to have anti-inflammatory effects and suppress PD-L1 expression. It may be a therapeutic strategy to include such herbal medicines to reduce the endogenous hormone effects on PD-L1 expression, which may interfere with chemotherapy. Signal transduction pathways involved in resveratrol suppress PD-L1 expression induced by thyroxine, as illustrated in Figure 1.
